# Long-term outcome in a person with pandrug-resistant HIV: the added value of a multidisciplinary approach

**DOI:** 10.1093/jacamr/dlae074

**Published:** 2024-05-16

**Authors:** Tommaso Clemente, Diana Canetti, Emanuela Messina, Elisabetta Carini, Liviana Della Torre, Rebecka Papaioannu Borjesson, Antonella Castagna, Vincenzo Spagnuolo

**Affiliations:** Infectious Diseases, IRCCS San Raffaele Scientific Institute, via Stamira d’Ancona, 20, 20127 Milan, Italy; Faculty of Medicine and Surgery, Vita-Salute San Raffaele University, Milan, Italy; Infectious Diseases, IRCCS San Raffaele Scientific Institute, via Stamira d’Ancona, 20, 20127 Milan, Italy; Infectious Diseases, IRCCS San Raffaele Scientific Institute, via Stamira d’Ancona, 20, 20127 Milan, Italy; Infectious Diseases, IRCCS San Raffaele Scientific Institute, via Stamira d’Ancona, 20, 20127 Milan, Italy; Infectious Diseases, IRCCS San Raffaele Scientific Institute, via Stamira d’Ancona, 20, 20127 Milan, Italy; Infectious Diseases, IRCCS San Raffaele Scientific Institute, via Stamira d’Ancona, 20, 20127 Milan, Italy; Faculty of Medicine and Surgery, Vita-Salute San Raffaele University, Milan, Italy; Infectious Diseases, IRCCS San Raffaele Scientific Institute, via Stamira d’Ancona, 20, 20127 Milan, Italy; Faculty of Medicine and Surgery, Vita-Salute San Raffaele University, Milan, Italy; Infectious Diseases, IRCCS San Raffaele Scientific Institute, via Stamira d’Ancona, 20, 20127 Milan, Italy

HIV multidrug resistance is associated with severely limited treatment options and a high risk of virological failure.^1^ New antiretroviral drugs offer a unique opportunity for people with MDR HIV (MDR-PWH) to achieve virological suppression,^2,3^ especially in a regimen containing ≥2 active molecules.^4,5^ If no drugs are effective according to genotypic resistance testing, other tools are needed to choose suppressive ART.

We describe a case of unexpected long-term virological suppression and immunological recovery despite no treatment options according to cumulative genotype, after using phenotypic resistance testing as part of a multidisciplinary approach to select optimized background therapy for combination with fostemsavir.

In May 2016, a >50-year-old man, followed at IRCCS San Raffaele Scientific Institute (Milan, Italy) since 2004, with HIV since 1995, had uncontrolled viral replication (HIV-RNA 1668 copies/mL, CD4+ 641 cells/mm^3^, CD4+/CD8+ 0.43), despite a regimen containing emtricitabine/tenofovir disoproxil fumarate 200/245 mg q24h, etravirine 200 mg q12h, darunavir/ritonavir 600/100 mg q12h and dolutegravir 50 mg q12h (Figure 1). He had no history of AIDS-defining events and his CD4+ nadir was 305 cells/mm^3^. He started ART in 1996 with a regimen containing azidothymidine, dideoxycytidine and (after 6 months) saquinavir; subsequently exposed to lamivudine, stavudine, didanosine, tenofovir disoproxil fumarate, emtricitabine, efavirenz, etravirine, indinavir, nelfinavir, fosamprenavir/ritonavir, atazanavir/ritonavir, darunavir/ritonavir, raltegravir, dolutegravir, enfuvirtide and maraviroc. His virological history was characterized by multiple failures mainly due to adherence issues with progressive development of resistance to the four major antiretroviral classes (he is currently enrolled in the PRESTIGIO Registry, an Italian multicentre cohort of MDR-PWH)^6^: in 2004 to nucleoside reverse transcriptase inhibitors, non-nucleoside reverse transcriptase inhibitors and protease inhibitors, in 2010 to integrase strand transfer inhibitors (Table S1, available as Supplementary data at *JAC-AMR* Online). His past medical history included arterial hypertension, lipoatrophy, dyslipidaemia, carotid stenosis, impaired fasting glucose, previous non-ST-elevation myocardial infarction (2011), previous hepatitis C virus [(HCV) positive anti-HCV antibodies] and hepatitis B virus [negative hepatitis B surface antigen (HBsAg), positive anti-HBsAg and anti-hepatitis B core antigen] infections; his comedications included rosuvastatin 20 mg q24h, metoprolol 25 mg q12h, acetylsalicylic acid 100 mg q24h.

**Figure 1.**
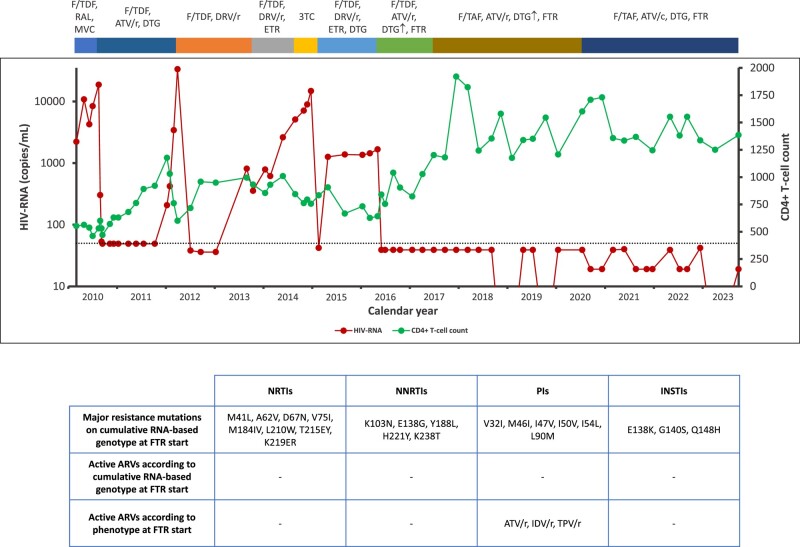
Virological and immunological outcome in the man with pandrug-resistant HIV since the first evidence of four-class drug resistance to the last visit. On the top: antiretroviral drug regimens since the first evidence of four-class drug resistance (March 2010) to the last visit. Graphic: HIV-RNA (red line) and CD4+ T-cell count (green line) trend represented by all the available values since the first evidence of four-class drug resistance to the last visit. On the bottom: major resistance mutations on cumulative RNA-based genotype at fostemsavir start (from the first genotype assessed in the history of the individual to the genotypic + phenotypic resistance test performed in April 2016), active antiretrovirals according to cumulative RNA-based genotype at fostemsavir start (according to Stanford HIV Drug Resistance Database, v.9.5), and active antiretrovirals according to phenotype at fostemsavir start (Phenosense GT plus Integrase by Monogram Biosciences, South San Francisco, CA, USA); from the left to the right NRTIs, NNRTIs, PIs and INSTIs. Abbreviations: ARV: antiretroviral; ATV/r: atazanavir/ritonavir 300/100 mg q24h; ATV/c: atazanavir/cobicistat 300/150 mg q24h; DRV/r: darunavir/ritonavir 600/100 mg q12h; DTG: dolutegravir 50 mg q12h; DTG↑: dolutegravir 100 mg q12h; ETR: etravirine 200 mg q12h; F/TAF: emtricitabine/tenofovir alafenamide 200/10 mg q24h; F/TDF: emtricitabine/tenofovir disoproxil fumarate 200/245 mg q24h; FTR: fostemsavir 600 mg q12h; IDV/r: indinavir/ritonavir 800/100 mg q12h; INSTI: integrase strand transfer inhibitor; NNRTI: non-nucleoside reverse transcriptase inhibitor; NRTI: nucleoside reverse transcriptase inhibitor; PI: protease inhibitor; TPV/r: tipranavir/ritonavir 500/200 mg q12h; 3TC: lamivudine 300 mg q24h.

With the availability of fostemsavir,^2,3^ genotypic resistance testing was repeated in December 2015: cumulative results showed pandrug resistance (Table S1). Tropism, resulting CXCR and combined genotypic + phenotypic resistance testing (Phenosense GT plus Integrase by Monogram Biosciences, South San Francisco, CA, USA) were performed in April 2016: phenotypically, the isolated viral strains were susceptible to atazanavir/ritonavir, indinavir/ritonavir and tipranavir/ritonavir (Figure 1 and Table S2). Based on these findings, ART was switched to emtricitabine/tenofovir disoproxil fumarate 200/245 mg q24h, atazanavir/ritonavir 300/100 mg q24h, dolutegravir 100 mg q12h and fostemsavir 600 mg q12h (BRIGHTE study, non-randomized cohort).^2,3^ The dose of rosuvastatin was reduced to 10 mg q24h, due to potential interactions with atazanavir/ritonavir and fostemsavir.

Since June 2016 (week 4 from fostemsavir initiation), the man has achieved and maintained virological suppression (HIV-RNA <50 copies/mL; Figure 1); during these 7 years, ART was simplified with dolutegravir dose reduction (50 mg q12h) after detection of a trough concentration of 15 843 ng/mL without associated symptoms, switches from emtricitabine/tenofovir disoproxil fumarate to emtricitabine/tenofovir alafenamide 200/10 mg q24h, and from atazanavir/ritonavir to atazanavir/cobicistat 300/150 mg q24h (Figure 1). During the follow-up, he developed type II diabetes mellitus, with a good glycaemic control through dietary measures.

After initiation of fostemsavir, an impressive immunological recovery was observed, with a CD4+ peak of 1920 cells/mm^3^ (December 2017). At the last visit, HIV-RNA was <50 copies/mL, CD4+ 1385 cells/mm^3^ and CD4+/CD8+ 0.76.

We reported a case of pandrug-resistant HIV infection in which a multidisciplinary approach, including phenotypic resistance testing for optimized background therapy selection and therapeutic drug monitoring (TDM), was essential to enhance fostemsavir efficacy and maintain favourable long-term virological and immunological outcomes.

The role of phenotypic resistance testing in predicting drug susceptibility is well established, although this technique evaluates only the circulating viral strains at the time of analysis and does not consider resistance archived in the viral reservoir; therefore, it could complement but not replace cumulative data from genotypic resistance testing.^7^ However, current HIV guidelines do not unanimously endorse its use in the diagnostic workflow, even in the absence of two fully active drugs according to genotype.^4,5^ This case supports that it could be a valuable resource, albeit difficult to access.

Moreover, TDM is not part of the routine management of suppressed people with HIV, except when drug–drug interactions are suspected.^4,5^ Nevertheless, it may be essential to tailor regimens in individuals with MDR HIV: the lack of therapeutic options might lead to the use of antiretrovirals at higher doses than commonly used, or to the combination of drugs to boost their plasma levels and activity, requiring close monitoring to both ensure achievement of therapeutic levels and to avoid potential toxicities. TDM could also be important to assess and improve adherence, allowing a safe reduction in pill burden where possible. It could be fundamental in case of comedications that may interact with antiretrovirals, given the impossibility of switching the ongoing regimen because of a too high risk of virological failure. All these aspects help to prevent the possible further accumulation of resistance. In the case described, despite intermediate resistance, dolutegravir was started at 100 mg q12h, also taking advantage of its potential interaction with atazanavir/ritonavir, supported by previous investigations in heavily treatment-experienced PWH.^8,9^ However, on detection of an abnormally high plasma concentration, the dose was promptly reduced to avoid toxicity and potential discontinuation.

Finally, in addition to virological suppression, temsavir has been suggested to play a role in immunological recovery,^3^ presumably through direct activity on gp120.^10^ Here, although the individual was not severely immunocompromised before starting fostemsavir, we believe that such an increase in CD4+ count (peak >3 times pre-fostemsavir value) could be unexpected, supporting a possible mechanism mediated by this compound, that needs to be fully elucidated.

In conclusion, the use of HIV phenotype and TDM is advisable for regimen selection and optimization in MDR-PWH, to achieve and maintain virological suppression and immunological recovery.

## Supplementary Material

dlae074_Supplementary_Data

## Data Availability

Anonymized data underlying this article will be shared on a reasonable request to the corresponding author.

## Transparency declarations

We declare no competing interests related to this report. D.C. has received research grants from Gilead Sciences and GlaxoSmithKline and payment for educational events and support for attending meetings from Merck Sharp & Dohme and ViiV Healthcare. A.C. has received personal fees for advisory boards, speaker panels and educational materials from Gilead Sciences, ViiV Healthcare, Janssen-Cilag, Merck Sharp & Dohme and Theratechnologies. V.S. has received grants from Gilead Sciences, personal fees for speaker panels from Gilead Sciences, ViiV Healthcare and Merck Sharp & Dohme. All other authors: no potential conflicts.

## Supplementary data

Tables S1 and S2 are available as Supplementary data at *JAC-AMR* Online.

## References

[1] Hsu RK, Fusco JS, Henegar CE et al Heavily treatment-experienced people living with HIV in the OPERA^®^ cohort: population characteristics and clinical outcomes. BMC Infect Dis 2023; 23: 91. 10.1186/s12879-023-08038-w 36782125 PMC9926692

[2] Kozal M, Aberg J, Pialoux G et al Fostemsavir in adults with multidrug-resistant HIV-1 infection. N Engl J Med 2020; 382: 1232–43. 10.1056/NEJMoa1902493 32212519

[3] Aberg JA, Shepherd B, Wang M et al Week 240 efficacy and safety of fostemsavir plus optimized background therapy in heavily treatment-experienced adults with HIV-1. Infect Dis Ther 2023; 12: 2321–35. 10.1007/s40121-023-00870-6 37751019 PMC10581994

[4] European AIDS Clinical Society . Guidelines. Version 12.0. https://www.eacsociety.org/guidelines/eacs-guidelines/.

[5] Panel on Antiretroviral Guidelines for Adults and Adolescents . Guidelines for the Use of Antiretroviral Agents in Adults and Adolescents with HIV. https://clinicalinfo.hiv.gov/en/guidelines/adult-and-adolescent-arv.

[6] Clemente T, Galli L, Lolatto R et al Cohort profile: PRESTIGIO, an Italian prospective registry-based cohort of people with HIV-1 resistant to reverse transcriptase, protease, and integrase inhibitors. BMJ Open 2024; 14: e080606. 10.1136/bmjopen-2023-080606 PMC10862296 38341206

[7] MacArthur RD . Understanding HIV phenotypic resistance testing: usefulness in managing treatment-experienced patients. AIDS Rev 2009; 11: 223–30. 19940949

[8] Ferrari D, Spagnuolo V, Manca M et al Increased dose of dolutegravir as a potential rescue therapy in multi-experienced patients. Antivir Ther 2019; 24: 69–72. 10.3851/IMP3275 30353884

[9] Spagnuolo V, Galli L, Poli A et al A nucleoside-sparing regimen of dolutegravir plus ritonavir-boosted atazanavir in HIV-1-infected patients with virological failure: the DOLATAV study. Drug Des Devel Ther 2019; 13: 477–9. 10.2147/DDDT.S192124 PMC6352870 30774311

[10] Richard J, Prévost J, Bourassa C et al Temsavir blocks the immunomodulatory activities of HIV-1 soluble gp120. Cell Chem Biol 2023; 30: 540–52.e6. 10.1016/j.chembiol.2023.03.003 36958337 PMC10198848

