# Supplementary material for: Long-term outcome in a person with pandrug-resistant HIV: the added value of a multidisciplinary approach
Source: JAC Antimicrob Resist. 2024 May 16;6(3):dlae074. doi: 10.1093/jacamr/dlae074 (PMC11098035; doi:10.1093/jacamr/dlae074)
Supplement: dlae074_Supplementary_Data [file dlae074_supplementary_data.docx]

**Long-term outcome in a person with pandrug-resistant HIV: the added value of a multidisciplinary approach**

**Tommaso CLEMENTE, Diana CANETTI, Emanuela MESSINA, Elisabetta CARINI, Liviana DELLA TORRE, Rebecka PAPAIOANNU BORJESSON, Antonella CASTAGNA, Vincenzo SPAGNUOLO**

**Table of Contents**

**Table S1**…………………………..………………………………………………………………………………………………………………………………………….2

**Table S2**…………………………..………………………………………………………………………………………………………………………………………….5

**Table S1.** Available genotypic data on antiretroviral drug resistance until December 2015.

|  | Cumulative data from RNA-based genotypic resistance tests pre-evidence of 4-class drug resistance^1^ | RNA-based genotypic resistance test at March 2010^1^ | RNA-based genotypic resistance tests at December 2014^1^ | RNA-based genotypic resistance test at  December 2015^1^ | Cumulative data from RNA-based genotypic resistance tests at December 2015^1^ |
| --- | --- | --- | --- | --- | --- |
| NRTI + NNRTI resistance-associated mutations | **NRTI:** M41L, D67N, V75I, M184I, L210W, T215EY, K219ER **- NNRTI:** K103N, Y188L, H221Y, K238T **- Other:** I31L, T39A, K43Q, V90I, K122E, S162Y, R172K, R211K | **NRTI:** M41L, D67N, V75I, M184I **- NNRTI:** K103N, Y188L **- Other:** I31L, T39A, K43Q, V90I, K122E, S162Y, R172K, V179I, G196E, T200A, E203D | **NRTI:** M41L, A62V, D67N, V75I, M184V, L210W, T215Y, K219R **- NNRTI:** K103N, E138G, Y188L, K238T **- Other:** I31L, T39A, K43Q, V90I, K122E, S162Y, R172K, V179I, G196E, T200A, E203D, R211K, D237N | **NRTI:** M41L, A62V, D67N, V75I, M184V, L210W, T215Y, K219R - **NNRTI:** K103N, E138G, Y188L, K238T - **Other:** I31L, T39A, K43Q, V90I, K122E, S162Y, R172K, V179I, G196E, T200A, E203D, R211K, D237N | **NRTI:** M41L, A62V, D67N, V75I, M184IV, L210W, T215EY, K219ER **- NNRTI:** K103N, E138G, Y188L, H221Y, K238T **- Other:** I31L, T39A, K43Q, V90I, K122E, S162Y, R172K, V179I, G196E, T200A, E203D, R211K, D237N |
| Abacavir (ABC) | High-level resistant | Intermediate resistant | High-level resistant | High-level resistant | High-level resistant |
| Didanosine (ddI) | High-level resistant | Intermediate resistant | High-level resistant | High-level resistant | High-level resistant |
| Emtricitabine (FTC) | High-level resistant | High-level resistant | High-level resistant | High-level resistant | High-level resistant |
| Lamivudine (3TC) | High-level resistant | High-level resistant | High-level resistant | High-level resistant | High-level resistant |
| Stavudine (d4T) | High-level resistant | **Low-level resistant** | High-level resistant | High-level resistant | High-level resistant |
| Zidovudine (AZT) | High-level resistant | **Low-level resistant** | High-level resistant | High-level resistant | High-level resistant |
| Tenofovir (TDF) | High-level resistant | **Susceptible** | High-level resistant | High-level resistant | High-level resistant |
| Efavirenz (EFV) | High-level resistant | High-level resistant | High-level resistant | High-level resistant | High-level resistant |
| Etravirine (ETR) | **Low-level resistant** | **Potentially resistant** | **Low-level resistant** | **Low-level resistant** | Intermediate resistant |
| Nevirapine (NVP) | High-level resistant | High-level resistant | High-level resistant | High-level resistant | High-level resistant |
| Rilpivirine (RPV) | High-level resistant | High-level resistant | High-level resistant | High-level resistant | High-level resistant |
| Doravirine (DOR)^2^ | High-level resistant | High-level resistant | High-level resistant | High-level resistant | High-level resistant |
| PI resistance-associated mutations | **Primary:** V32I, I47V, I50V, L90M **- Accessory:** none **- Other:** L10I, I13V, L19I, K20R, E35D, M36I, L63P, A71V, I85V | **Primary:** V32I, I47V, I50V, L90M **- Accessory:** none **- Other:** L10I, I13V, L19I, K20R, E35D, M36I, L63P, I66F, A71V, V82I, I85V | **Primary:** V32I, M46I, I47V, I50V, I54L, L90M **- Accessory:** L33F **- Other:** L10I, I13V, G16E, L19I, K20R, E35D, M36I, P39S, K55R, L63P, I66F, K70E, A71V, V82I, I85V, Q92R | **Primary:** V32I, M46I, I47V, I50V, I54L, L90M **- Accessory:** L33F **- Other:** L10I, I13V, G16E, L19I, K20R, E35D, M36I, P39S, L63P, I66F, A71V, V82I, I85V, Q92R | **Primary:** V32I, M46I, I47V, I50V, I54L, L90M **- Accessory:** L33F **- Other:** L10I, I13V, G16E, L19I, K20R, E35D, M36I, P39S, K55R, L63P, I66F, K70E, A71V, V82I, I85V, Q92R |
| Atazanavir/ritonavir (ATV/r) | Intermediate resistant | Intermediate resistant | High-level resistant | High-level resistant | High-level resistant |
| Darunavir/ritonavir (DRV/r) | Intermediate resistant | Intermediate resistant | High-level resistant | High-level resistant | High-level resistant |
| Fosamprenavir/ritonavir (FPV/r) | High-level resistant | High-level resistant | High-level resistant | High-level resistant | High-level resistant |
| Indinavir/ritonavir (IDV/r) | High-level resistant | High-level resistant | High-level resistant | High-level resistant | High-level resistant |
| Lopinavir/ritonavir (LPV/r) | High-level resistant | High-level resistant | High-level resistant | High-level resistant | High-level resistant |
| Nelfinavir (NFV) | High-level resistant | High-level resistant | High-level resistant | High-level resistant | High-level resistant |
| Saquinavir/ritonavir (SQV/r) | High-level resistant | High-level resistant | High-level resistant | High-level resistant | High-level resistant |
| Tipranavir/ritonavir (TPV/r) | Intermediate resistant | Intermediate resistant | Intermediate resistant | Intermediate resistant | Intermediate resistant |
| INSTI resistance-associated mutations | **Never assessed** | **Primary:** G140S, Q148H **- Accessory:** none **- Other:** E10D, M154I, V165I, V201I, I208L | **Primary:** G140S, Q148H **- Accessory:** none **- Other:** D3E, E10D, M154I, V165I, V201I, I208L | **Primary:** G140S, Q148H **- Accessory:** none **- Other:** D3E, E10D, M154I, V165I, V201I, I208L | **Primary:** G140S, Q148H **- Accessory:** none **- Other:** D3E, E10D, M154I, V165I, V201I, I208L |
| Dolutegravir (DTG) | - | Intermediate resistant | Intermediate resistant | Intermediate resistant | Intermediate resistant |
| Elvitegravir (EVG) | - | High-level resistant | High-level resistant | High-level resistant | High-level resistant |
| Raltegravir (RAL) | - | High-level resistant | High-level resistant | High-level resistant | High-level resistant |
| Bictegravir (BIC)^2^ | - | Intermediate resistant | Intermediate resistant | Intermediate resistant | Intermediate resistant |
| Cabotegravir (CAB)^2^ | - | High-level resistant | High-level resistant | High-level resistant | High-level resistant |

No available data on fusion inhibitors but history of virological failure and drug intolerance during enfuvirtide-containing regimens (2009).

^1^Prediction according to Stanford HIV Drug Resistance Database (version 9.5, hivdb.stanford.edu)

^2^Still not available in May 2016.

**Table S2.** Results of the combined genotypic + phenotypic resistance testing performed at April 2016.

| Combined genotypic + phenotypic resistance testing (April 2016)^1^ | | | | |
| --- | --- | --- | --- | --- |
|  | **Genotype** | **Phenotype** | **Cutoffs (Lower-Upper)^2^** | **Fold Change^3^** |
| NRTI + NNRTI resistance-associated mutations | **NRTI:** M41L, A62V, D67N, V75I, M184V, L210W, T215Y, K219R  **NNRTI:** V90I, K103N, E138G, V179I, Y188L, K238T | | | |
| Abacavir (ABC) | Resistant | **Partially sensitive** | 4.5 - 6.5 | 5.72 |
| Didanosine (ddI) | Resistant | **Partially sensitive** | 1.3 - 2.2 | 1.49 |
| Emtricitabine (FTC) | Resistant | Resistant | 3.5 | >MAX |
| Lamivudine (3TC) | Resistant | Resistant | 3.5 | >MAX |
| Stavudine (d4T) | Resistant | Resistant | 1.7 | 1.81 |
| Zidovudine (AZT) | Resistant | Resistant | 1.9 | 17 |
| Tenofovir (TDF) | Resistant | **Partially sensitive** | 1.4 - 4 | 1.98 |
| Delavirdine (DLV) | Resistant | Resistant | 6.2 | >MAX |
| Efavirenz (EFV) | Resistant | Resistant | 3 | >MAX |
| Etravirine (ETR) | Resistant | Resistant | 2.9 - 10 | 81 |
| Nevirapine (NVP) | Resistant | Resistant | 4.5 | >MAX |
| Rilpivirine (RPV) | Resistant | Resistant | 2 | >MAX |
| PI resistance-associated mutations | L10I, V11I, I13V, K20R, V32I, L33F, E35D, M36I, M46I, I47V, I50V, I54L, A71V, V82I, I85V, L90M | | | |
| Atazanavir (ATV) | Resistant | Resistant | 2.2 | 2.99 |
| Atazanavir/ritonavir (ATV/r) | Resistant | **Sensitive** | 5.2 | 2.99 |
| Darunavir/ritonavir (DRV/r) | Resistant | Resistant | 10 - 90 | >MAX |
| Fosamprenavir/ritonavir (FPV/r) | Resistant | Resistant | 4 - 11 | >MAX |
| Indinavir/ritonavir (IDV/r) | Resistant | **Sensitive** | 10 | 2.37 |
| Lopinavir/ritonavir (LPV/r) | Resistant | Resistant | 9 - 55 | 90 |
| Nelfinavir (NFV) | Resistant | Resistant | 3.6 | 13 |
| Ritonavir (RTV) | Resistant | Resistant | 2.5 | >MAX |
| Saquinavir/ritonavir (SQV/r) | Resistant | **Partially sensitive** | 2.3 - 12 | 7.7 |
| Tipranavir/ritonavir (TPV/r) | **Sensitive** | **Sensitive** | 2 - 8 | 0.52 |
| INSTI resistance-associated mutations | T97A, E138K, G140S, Q148H | | | |
| Dolutegravir (DTG) | Resistant | Resistant | 4 - 13 | >MAX |
| Elvitegravir (EVG) | Resistant | Resistant | 3.5 | >MAX |
| Raltegravir (RAL) | Resistant | Resistant | 2.2 | >MAX |

^1^Phenosense GT plus Integrase by Monogram Biosciences (South San Francisco, CA)

^2^Lower clinical cutoff defined as the fold change which is the best discriminator of reduced clinical response. Upper clinical cutoff defined as the fold change above which a clinical response is unlikely (<0.5 log copies/mL of reduction in HIV RNA). Biological cutoffs used for AZT, NNRTIs and non-ritonavir-boosted PIs, and defined as the fold change below which reside 99% of tested wild-type isolates.

^3^Fold change defined as the ratio between the concentration of drug required to inhibit viral replication by 50% (IC50) in the individual and IC50 of reference.
